# Global genomic surveillance of *Salmonella* in the environment: assessing virulence and antimicrobial resistance at scale

**DOI:** 10.1128/mbio.01142-26

**Published:** 2026-06-22

**Authors:** Viraj Bandsode, Shamsul Qumar, Anuradha Singh, Debaleena Das, Humera Quadriya, Mahanga Nyambero, Vikas Gawai, Ayan Mahapatra, Torsten Semmler, Pittu Sandhya Rani, Niyaz Ahmed

**Affiliations:** 1 Pathogen Biology Laboratory, Department of Biotechnology, and Bioinformatics, University of Hyderabad, Hyderabad, Telangana, India; 2 University of Sharjah, Sharjah Institute for Medical Research, Sharjah, United Arab Emirates; 3 Robert Koch Institute 9222, Berlin, Germany; Universiteit Gent, Gent, Belgium

**Keywords:** environmental *Salmonella*, antimicrobial resistance, whole-genome sequencing, plasmidome, resistome, One Health

## Abstract

*Salmonella* is a globally distributed zoonotic pathogen with widespread environmental persistence; however, genomic characterization of environmental isolates from underrepresented regions remains limited. Current global data sets are predominantly populated with genomes from high-income countries, restricting our ability to resolve evolutionary trajectories, ecological adaptations, and emerging antimicrobial resistance (AMR). We performed a comparative genomic analysis of 1,399 high-quality *Salmonella* genomes, integrating 54 newly sequenced isolates from India (representing surface water and soil samples) with global data sets. Phenotypic analysis showed that 55.6% of the Indian isolates were multidrug-resistant, and 72.2% displayed strong biofilm-forming capacity. Integration of global genomes revealed extensive phylogenetic interspersion, reflecting widely distributed lineages shaped by shared ancestry or environmentally mixed *Salmonella* populations. The pangenome comprised 20,915 genes, with a 3,394 core, and a large accessory genome (>16,001 cloud genes). Serogroups B and C2-C3 dominated globally and carried the broadest AMR repertoires. While efflux-associated and regulatory resistance genes were conserved across subspecies, acquired determinants such as aminoglycoside-modifying enzymes, *tet(A/B)*, *sul* genes, and rare extended-spectrum β-lactamases (ESBLs) varied by serogroup. Detection of *mcr-1*, *mcr-5*, and *mcr-9* highlights early circulation of colistin resistance in environmental reservoirs. Core virulence loci (SPI-1/SPI-2) remained uniformly conserved, whereas accessory modules, including *spv* and *pef* operons, siderophore systems (*iro*, *iuc/iut*), and stress-response genes, showed serogroup-specific enrichment. Plasmidome analysis revealed marked diversity, dominated by IncF and colicinogenic plasmids, with serogroup-specific patterns, suggesting niche adaptation and horizontal gene transfer. Overall, environmental *Salmonella* constitute a globally connected and genetically dynamic reservoir where conserved virulence backbones coexist with rapidly evolving resistance and plasmid repertoires. These findings position environmental surveillance as a cornerstone of One Health preparedness for tackling high-risk, pathogenic lineages of *Salmonella*.

**IMPORTANCE:**

*Salmonella* inhabiting environmental niches, such as water and soil, remain underexplored despite their potential role in pathogen gene pool evolution and infection burden. Using a global data set that includes newly sequenced genomes of isolates from India, we show that environmental populations are active evolutionary reservoirs that maintain a conserved virulence core while rapidly exchanging antimicrobial resistance genes via horizontal gene transfer. The detection of early-stage colistin resistance and multidrug-resistant lineages in global ecosystems identify these environments as potential early-warning systems for emerging clinical threats. Our findings demonstrate that Indian environmental strains of *Salmonella* are deeply interconnected with global lineages, underscoring the need for global surveillance. Collectively, genomic epidemiology as described herein reinforces a One Health framework and highlights environmental surveillance as a critical requirement in the context of high-risk pathogens such as *Salmonella*.

## INTRODUCTION

*Salmonella* is a leading cause of gastroenteritis worldwide, with substantial morbidity across human and animal populations (1–3). The species *Salmonella enterica* comprises over 2,600 serovars, broadly categorized as typhoidal and non-typhoidal (3). While typhoidal serovars are host-restricted, non-typhoidal *Salmonella* (NTS) serovars infect a broad range of hosts, making them more prevalent, highly transmissible, and difficult to control (4). NTS accounts for a substantial burden of *Salmonella*-related infections in humans and animals globally, with an estimated 93.8 million infections and 155,000 fatalities occurring annually (5, 6). NTS infections also exert substantial global economic costs, with the highest impact observed in Sub-Saharan Africa among vulnerable populations, including children, elderly individuals, and those living with HIV (1, 7, 8). Beyond treatment costs, NTS significantly affects productivity and healthcare systems, particularly in resource-constrained developing regions. The cumulative effect results in a major drain on global wealth, impeding economic growth and placing long-term pressures on public health infrastructure worldwide (9).

Environmental pathogens are often considered particularly concerning because they are not constrained by host survival and can therefore maintain high virulence while persisting for extended periods in soil or water, even in the absence of viable host populations. Furthermore, their inherent resilience to external conditions makes them naturally resistant to many antimicrobial substances and nearly impossible to eradicate from the environment. While typhoidal and non-typhoidal strains associated with human diseases are well-studied, much less is known about the diversity, ecology, and evolutionary dynamics of isolates in environmental reservoirs (10, 11). These natural ecosystems, including surface water, soil, agricultural interfaces, and emerging contaminants, constitute critical but understudied components of the bacterial transmission cycle (12, 13). Environmental reservoirs act as dynamic niches, where pathogens can persist, adapt, and accumulate antimicrobial resistance (AMR) and virulence factors, thereby leading to spillovers to human and animal populations (14, 15).

The environmental persistence of multidrug-resistant (MDR) *Salmonella* and its interspecies transmission is prominent. *Salmonella* frequently harbors MDR strains enriched with efflux pumps, mobile genetic elements (MGEs), plasmid-borne resistance genes, and stress-adaptation mechanisms (16). Such reservoirs play a pivotal role in shaping pathogen evolution through horizontal gene transfer (HGT) and biofilm-mediated survival. Considering the risks associated with environmental *Salmonella*, the global genomic representation remains sparse and geographically skewed. Insubstantial genomic representation in public databases from middle- and low-income countries limits the understanding of AMR trajectories, serogroup diversity, and plasmid dynamics (17, 18).

Using whole-genome sequencing (WGS), we sought to harness platform abilities to resolve pathogen diversity and thereby enable fine-scale characterization of global *Salmonella* lineages, resistomes, virulomes, and mobilomes (19). We selectively isolated *Salmonella* from environmental niches of Indian origin, sequenced their genomes, and integrated them with a curated global data set to expand the scope of comparative genomics. Leveraging the expanded data set enabled robust insights into the pangenome architecture, phylogenetic relationships, serogroup distribution, resistome-virulome profiles, and mobilome diversity. This large-scale genomic investigation elucidates the evolutionary dynamics, ecological adaptations, and public health relevance of genomic epidemiology for strengthening One Health-guided surveillance of environmental *Salmonella*.

## RESULTS

### Phenotypic characterization and genomic profiles of fresh isolates

**Fig 1:**
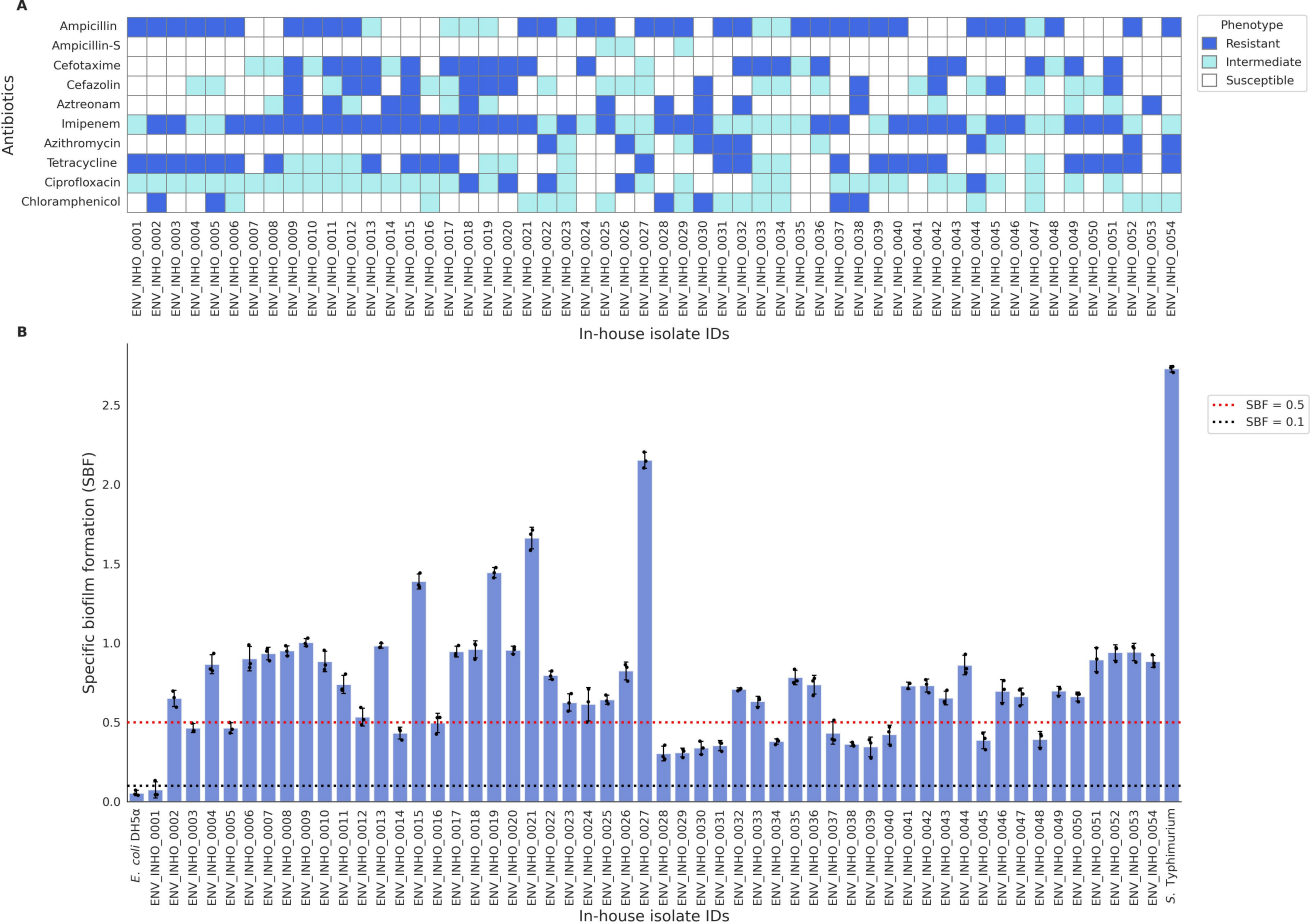
Antibiotic resistance profiles and biofilm formation capabilities of newly sequenced environmental isolates. (**A**) Heatmap depicting the antibiotic susceptibility phenotypes of 54 newly sequenced isolates (ENV_INHO_0001 to ENV_INHO_0054) against ten different antimicrobial agents: the intensity of blue color corresponds to the resistance phenotypes of the isolates—resistant (dark blue), intermediate (light blue), and susceptible (white). The panel includes beta-lactams (ampicillin, cefotaxime, cefazolin, and imipenem), aminoglycosides, fluoroquinolones and others, as indicated on the left, revealing distinct MDR patterns across the data set. (**B**) Quantification of specific biofilm formation (SBF) for the corresponding newly sequenced isolates: biofilm formation was assessed via crystal violet staining, and SBF values presented as the mean ± standard deviation. The horizontal red dashed line (SBF = 0.5) denotes the threshold for strong biofilm formation, while the black dashed line (SBF = 0.1) indicates the baseline cutoff. *E. coli* DH5α served as the negative control, and *S. enterica* subsp. *enterica* serovar Typhimurium (ATCC 14028) as the positive control. High variance was observed with isolates such as ENV_INHO_0027 exhibiting hyper-biofilm forming phenotypes (SBF > 2.0).

Environmental samples from soil and water in India yielded 54 *Salmonella* isolates identified by selective culture, biochemical characterization, and genus-specific PCR amplification of the *invA* gene. Among these, 40 isolates originated from water and 14 from soil, corresponding to an overall prevalence of 10.67%. All these isolates (*n* = 54) were subjected to antimicrobial susceptibility testing (AST) against 10 standard antibiotics representing nine classes. The majority of isolates exhibited resistance against at least one antibiotic, except ampicillin-sulbactam, while 55.56% of the isolates were classified as MDR (Fig. 1A). Among all antibiotics tested, ampicillin-sulbactam appeared to be the most effective, with almost all isolates showing susceptibility and only three (ENV_INHO_0025, ENV_INHO_0026, and ENV_INHO_0029) exhibiting intermediate resistance (5.6%). Among β-lactam antibiotics, the highest rates of resistance were observed to imipenem (62.96%) and ampicillin (57.41%), followed by cefotaxime (37.04%), cefazolin (22.22%), and aztreonam (20.37%). In contrast, lower resistance frequencies were recorded for azithromycin (14.81%), chloramphenicol (11.11%), and ciprofloxacin (9.26%). Notably, a considerable proportion of isolates exhibited intermediate resistance to ciprofloxacin (62.9%) and chloramphenicol (30%), suggesting a gradual shift toward reduced susceptibility to these antibiotics (Fig. 1A).

Biofilm formation was observed in all in-house isolates, at varying levels. A majority of the isolates were classified as strong biofilm formers (72.2%), followed by moderate formers (25.92%), with only a single isolate (1.9%) demonstrating weak biofilm formation (Fig. 1B). Among the in-house isolates, ENV_INHO_0019, ENV_INHO_0021, and ENV_INHO_0027 exhibited the highest biofilm-forming capacity, with specific biofilm formation (SBF) values comparable to that of the positive control, whereas ENV_INHO_0001 showed the weakest activity.

All in-house isolates (*n* = 54), as mentioned above, were subsequently subjected to WGS and *de novo* assembly. Quality assessment with different tools (see Materials and Methods section) revealed high-quality draft genomes, with an average genome size of 4.7 Mbp and a mean GC content of 52.16%. The BioSample and sequence-associated information for all the genomes is summarized in Tables S1 (genomes of clinical strains) and S2 (all other genomes) (https://github.com/Viraj-105/Environmental_Salmonella_Genomics).

Whole-genome sequencing of 54 newly sequenced Indian environmental *Salmonella* isolates revealed an enrichment of AMR determinants primarily driven by efflux systems and global regulators. All isolates (100%) encoded a complete intrinsic RND efflux backbone (*acrAB-tolC*), as well as *mdsABC* and *mdtBC,* indicating a genetic potential for strong antimicrobial extrusion. Additional transporters (*mdtK*, *emrAB*, and *yojI*) and *msbA* further expand this repertoire, while key regulators (*marA*, *baeR*, *cpxA*, *KdpE*, *golS*, *sdiA*, and *H-NS*) highlight coordinated control of resistance and stress responses. The presence of *bacA* and *ampH* suggests supplementary mechanisms, including peptide tolerance and β-lactam-associated activity, collectively portraying a highly integrated, efflux-dominated resistome in *Salmonella*.

Virulence analysis revealed substantial pathogenic potential, with universal conservation of key virulence factors including SPI-2 (*ssa/sse*), the *csg* fimbrial system, and iron acquisition systems (*ent/fep*). Notably, almost all the isolates also harbored SPI-1(*inv*) and the *spvBCR* locus, indicating genetic capacity for host invasion and systemic infection.

The plasmidome was dominated by a highly conserved IncFII(S)/IncFIB(S) replicon signature (98.1%), consistent with the *S. enteritidis* virulence plasmid (pSEN), and uniformly maintained across both soil and water niches, suggesting strong ecological stability.

### Global data set for comparative genomics

**Fig 2:**
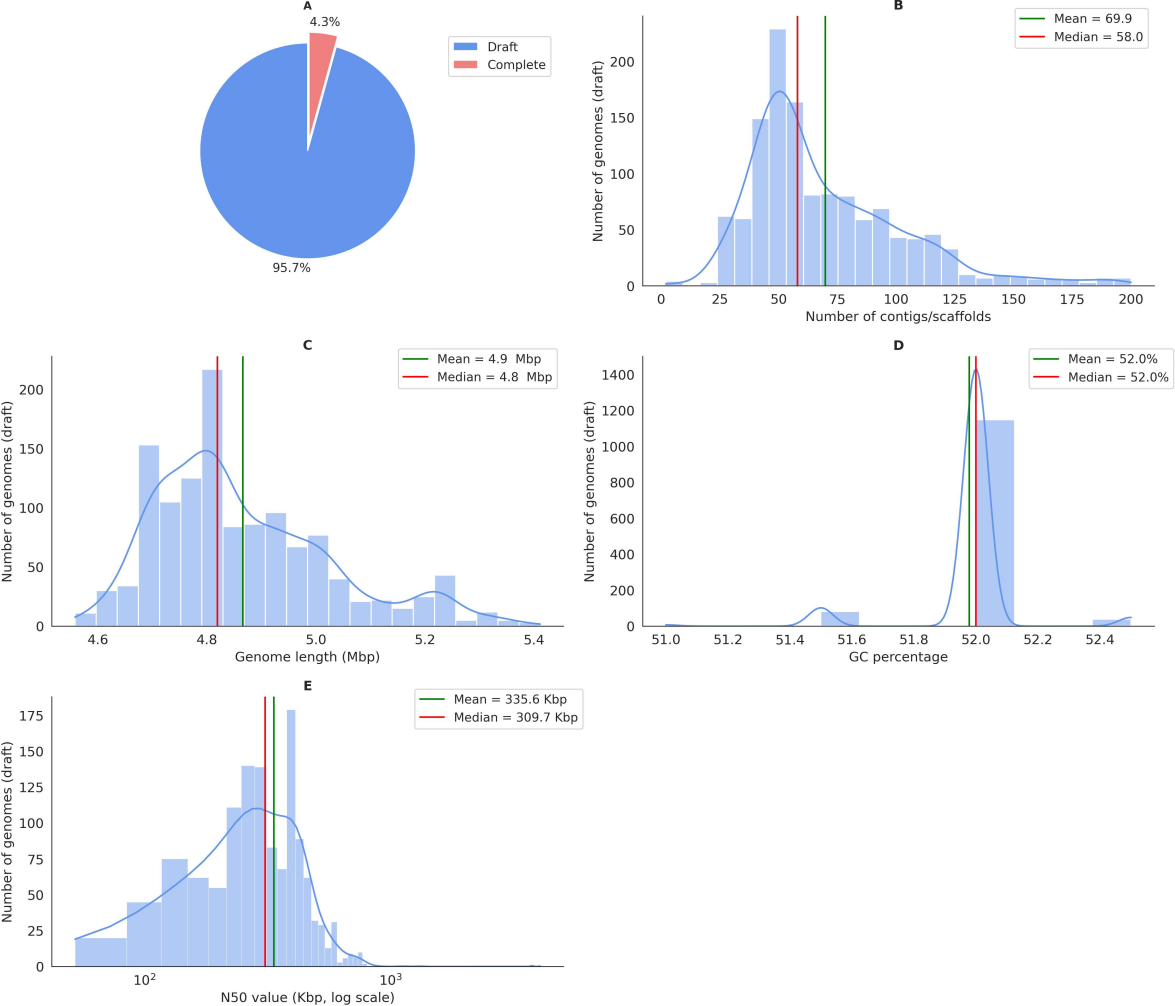
Genome assembly statistics of open source data set. (**A**) Pie chart representing draft and complete genome assembly status across the data set. Panels B–E show histograms (see details below) summarizing the key quality metrics for the draft genomes containing fewer than or equal to 200 contigs. In each panel, the blue curve represents the kernel density estimation, the vertical green line denotes the mean, and the vertical red line denotes the median. (**B**) Distribution of fragmentation levels was measured by the number of contigs/scaffolds per genome. (**C**) Distribution of total genome length in mega-base pairs (Mbp): the genomes clustered around a median size of 4.8 Mbp (mean = 4.9 Mbp), typical of *Salmonella* genomes. (**D**) GC content distribution: the data set exhibits high uniformity with a sharp peak at 52.0% (mean and median), indicating taxonomic consistency. (**E**) Assessment of assembly contiguity using N50 values (kbp, log scale): the distribution shows a median N50 of 309.7 kbp (mean = 335.6 kbp), suggesting reasonably high contiguity for short-read assemblies.

The sequence data of in-house *Salmonella* isolates were seamlessly integrated into a larger collection of 1,333 high-quality *Salmonella* genomes representing diverse geographic regions and climate conditions (Table S2: https://github.com/Viraj-105/Environmental_Salmonella_Genomics), resulting in a comprehensive data set of 1,387 environmental *Salmonella* genomes. Among the 1,333 open-source genomes, 4.3% (*n* = 57) were complete, and 95.7% (*n* = 1,276) were draft assemblies. The draft genomes contained fewer than 200 contigs and had an N50 of at least 52,240 bp, an average genome size of 4.9 Mbp, and an average GC content of ~52% (Fig. 2).

A total of 1,387 environmental genomes, including 54 in-house genomes from India, were compiled for comparative genomic analyses. The geographic distribution of this collection spanned 25 countries, dominated by genomes from the United States (67.77%, *n* = 940), followed by South Africa (5.34%, *n* = 74), Mexico (5.19%, *n* = 72), Canada (4.25%, *n* = 59), India (3.89%, *n* = 54), and Singapore (3.39%, *n* = 47). Each of the remaining countries contributed fewer than 30 genomes, and five of those genomes lacked the associated country metadata. These genomes represented diverse environmental niches, including pond water, river water, wastewater, and farm-environment swabs. Overall, the majority were recovered from aquatic sources, with a smaller proportion originating from soil. Inclusion of 12 well-characterized clinical reference strains expanded the final data set to 1,399 genomes. This data set, encompassing environmental isolates from across the globe and clinical reference strains, was used for downstream comparative genomic analyses.

### Pangenome architecture and population structure of environmental *Salmonella*

Pangenome analysis identified 20,915 orthologous genes across the data set, highlighting the genomic diversity of *Salmonella*. Out of these, 3,394 genes constituted the core genome (present in ≥99% of isolates), 278 were classified as soft-core (95%–99%), 1,242 as shell genes (15%–95%), and 16,001 as cloud genes (<15%). Genomic plasticity, with the dominance of accessory genes, indicates that environmental *Salmonella* possess an open pangenome.

**Fig 3:**
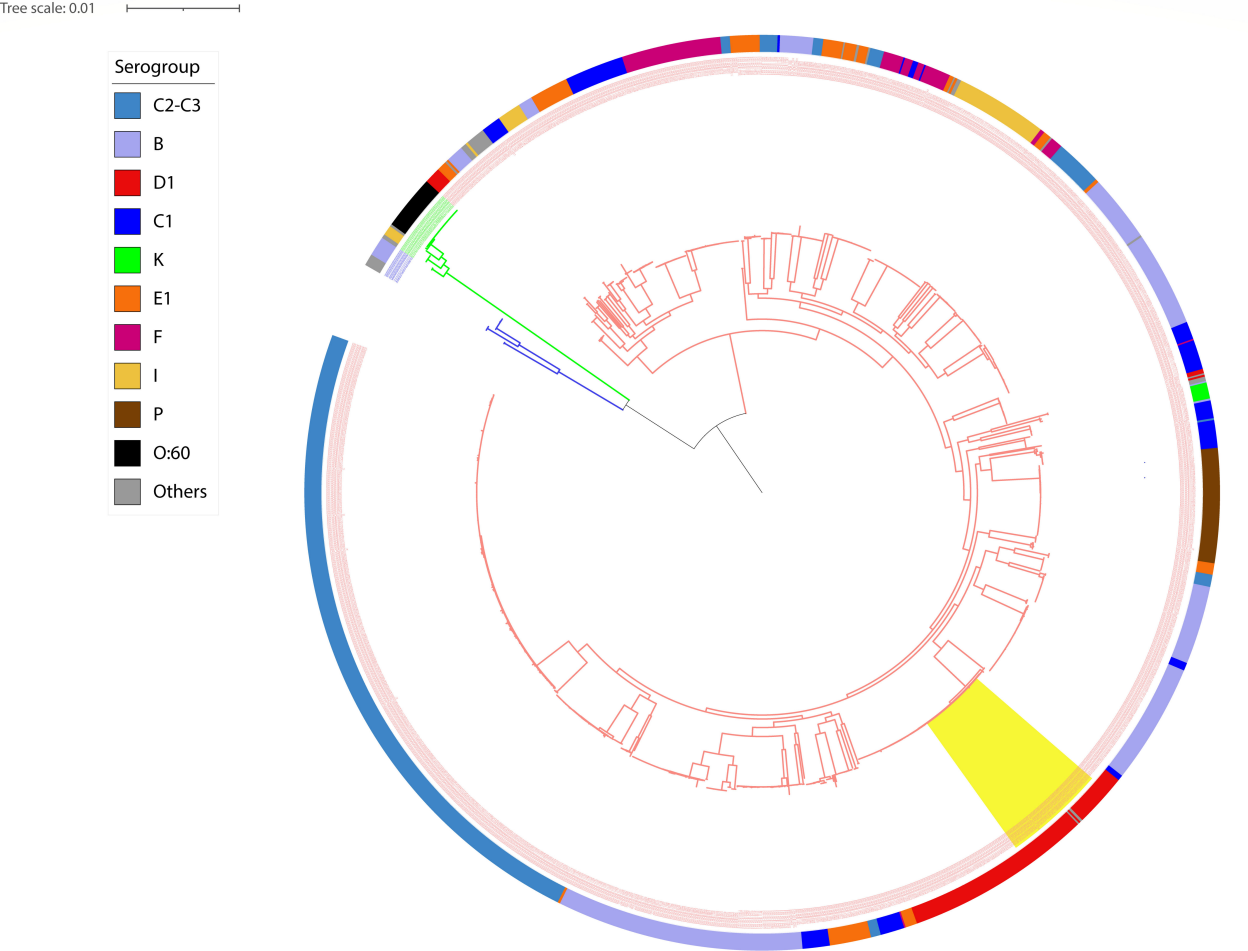
Phylogenetic structure of the *Salmonella* genome collection in the study comprised of a maximum-likelihood tree of 1,399 genomes based on 3,394 concatenated core gene alignment. The phylogeny resolves three major clades corresponding to *S. enterica* subsp. *enterica*, *S. enterica* subsp. *diarizonae* and *S. enterica* subsp. *salamae*. Colored outer ring of serogroup diversity demonstrates lineage-associated clustering patterns. The population is structured into three dominant, highly clonal sub-clades corresponding to serogroups C2-C3 (medium blue), B (light blue), and D1 (red). In contrast, serogroups K (green) and O:60 (black) form deeply branching divergent lineages distinct from the primary *S. enterica* clusters, suggesting phylogenetically divergent lineages. The yellow-shaded area highlights the cluster containing the newly sequenced isolates.

A maximum-likelihood phylogeny of the core genes revealed substantial phylogenetic diversity and resolved the collection into three major clades corresponding to distinct *Salmonella* subspecies (Fig. 3). The largest clade comprised *S. enterica* subsp. *enterica*, while the remaining two clades comprised *Salmonella diarizonae* and *Salmonella salamae*, respectively. All 54 in-house isolates clustered exclusively within the *S. enterica* subsp. *enterica* clade. Within this clade, the phylogeny revealed a composite population structure. A large, densely clustered group of closely related genomes suggests shared ancestry, whereas multiple branched, low-diversity subclades reflect long-term evolutionary divergence. Importantly, serogroups did not segregate into discrete phylogenetic clusters but were instead distributed across multiple subclades, underscoring the complex evolutionary dynamics of antigenic variation in *Salmonella*.

Projection of serogroup metadata alongside the phylogeny revealed marked heterogeneity. Serogroups C2-C3 and B formed the most prominent and contiguous phylogenetic clusters, whereas serogroups C1, D1, E1, K, and F were dispersed across multiple subclades, consistent with frequent recombination, HGT, or serogroup switching events. In contrast, serogroup P formed a distinct monophyletic cluster, suggesting a genetically coherent lineage potentially shaped by niche adaptation. Similarly, isolates belonging to the O:60 serogroup clustered tightly within a restricted phylogenetic branch, consistent with recent emergence or limited dissemination. Miscellaneous serogroups were broadly scattered across the tree and did not form dominant or cohesive clades (Fig. 3).

Genomic typing further corroborated these phylogenetic patterns. *S. enterica* subsp. *enterica* was the dominant subspecies (*n* = 1,347), followed by *S. diarizonae* (*n* = 36) and *S. salamae* (*n* = 16). Environmental genomes were most frequently associated with serovars Newport (*n* = 264), Enteritidis (*n* = 122), and Saintpaul (*n* = 80), whereas clinical reference genomes (*n* = 12) included Typhimurium, Typhi, Paratyphi A, B (var. Java), and C.

**Fig 4:**
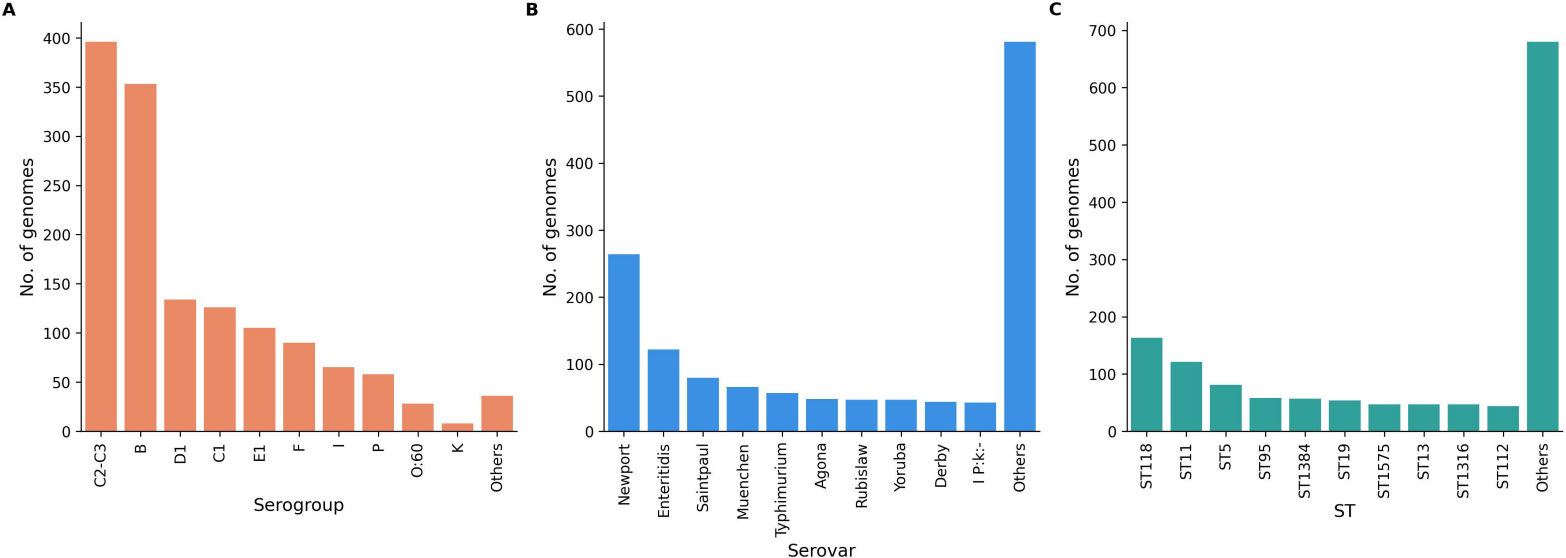
Population structure and molecular typing distribution of the *Salmonella* data set. (**A**) Serogroup diversity: within the genome collection, the data set was dominated by serogroups C2-C3 and B, with serogroups D1, C1, and E1 also represented in significant numbers. (**B**) Serovar diversity—distribution of the top 10 identified serovars: *S*. Newport and *S*. Enteritidis were the most prevalent individual serovars. (**C**) Sequence type (ST) diversity: genotypic classification based on multilocus sequence typing (MLST) profiles portrayed a polyclonal population with numerous low-frequency sequence types.

Across the whole collection, 116 distinct multilocus sequence types (STs) were identified, with more than half represented by fewer than ten genomes, indicating substantial strain-level diversity. The most prevalent STs, ST118, ST11, and ST5, together accounted for over 25% of the data set, reflecting the global dominance of specific high-fitness strains. Strong concordance between serogroups, serovars, and STs was observed, including serogroup B with *S*. Typhimurium (ST19) and *S*. Saintpaul (ST95), serogroup C2-C3 with *S*. Newport (ST118 and ST5), and serogroup D1 with *S*. Enteritidis (ST11). Rare serogroups and STs formed small, phylogenetically distinct clusters, further emphasizing the breadth of genomic diversity captured within this collection (Fig. 4).

### Whole genome-based resistome profiling

The genomic data set was screened for genetic elements associated with AMR, revealing a complex landscape reflecting diverse evolutionary patterns and selective pressures across subspecies and serogroups. Plasmids were found to be the major carriers of AMR genes (Table S3: https://github.com/Viraj-105/Environmental_Salmonella_Genomics), as described in detail in the later part.

**Fig 5:**
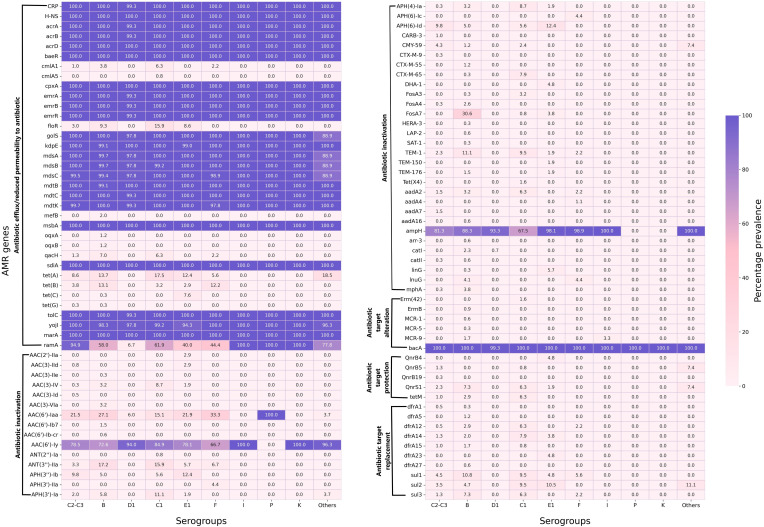
Heatmap showing the percentage prevalence of AMR genes categorized by mechanism of action. Serogroups are displayed on the *x*-axis, while AMR determinants are clustered on the *y*-axis by functional classes—antibiotic efflux/reduced permeability, antibiotic inactivation, target alteration, target protection, and target replacement. The color gradient represents percent prevalence within each serogroup, ranging from 0% (pink) to 100% (purple).

The prevalence of AMR genes varied across the three *Salmonella* subspecies, with many genes highly conserved across the data set. Figure S1 (https://github.com/Viraj-105/Environmental_Salmonella_Genomics) highlights genes present in *S. salamae* or *S. diarizonae* that are also found in *S. enterica*. Given the considerably larger gene repertoire of *S. enterica*, its comprehensive AMR profile is detailed separately (Fig. 5). A set of intrinsic resistance-associated determinants, including *acrA*, *acrB*, *acrD*, *baeR*, *cpxA*, *emrABR*, *hns*, *marA*, *mdtBC*, *msbA*, *sdiA*, and *tolC,* was detected in 99%–100% of *S. enterica*, *S. diarizonae*, and *S. salamae* genomes, representing conserved multidrug efflux systems, global regulators, and housekeeping functions. However, a subset of genes exhibited marked subspecies-specific variation. AMR determinants were non-randomly distributed across *S. enterica* serogroups, with enrichment analysis identifying 66 significant gene-lineage associations (false discovery rate [FDR] < 0.05) (Table S4: https://github.com/Viraj-105/Environmental_Salmonella_Genomics). Notably, *ampH*, *golS*, *mdsA*, *mdsB*, *mdsC*, and *mdtK* were completely absent in *S. diarizonae* but present in the majority of the *S. enterica* and *S. salamae* genomes. Similarly, *aac(6')-Iy* showed moderate prevalence in *S. enterica* (76.4%) but was detected in 100% of *S. diarizonae* and *S. salamae* genomes. The *ampH* gene also displayed variation, being present in 82.7% of *S. enterica* and 62.5% of *S. salamae* but absent in *S. diarizonae*. In contrast, *yojI* was nearly ubiquitous in *S. enterica* (98.7%) and *S. diarizonae* (100%) but occurred in only 25% of *S. salamae*. Comparative analysis across subspecies revealed lineage-specific patterns. *S. diarizonae* notably lacked several efflux-related genes (*ampH*, *golS*, *mdsABC*, and *mdtK*), suggesting potential gene loss or divergence within this subspecies. Comparative analysis of AMR genes across *S. enterica* serogroups revealed diverse prevalence patterns.

Notably, 44.55% of the detected genes (*n* = 101) were associated with antibiotic inactivation, 34.65% with antibiotic efflux, and the remaining 20.79% with antibiotic target alteration. The percentage prevalence of AMR genes across serogroups is further illustrated in Fig. 5, focusing on the nine major serogroups of *S. enterica*.

Efflux pump-associated genes, global regulatory elements, and core functional resistance determinants together constituted the conserved component of the resistome, with prevalence across all serogroups, consistent with previous reports. Intrinsic multidrug efflux systems (e.g., *acrAB/acrD, emrAB, mdtBC, tolC, msbA,* and *yojI*), global regulators (*marA, ramA, sdiA, baeR,* and *cpxA*), and core functional genes (*ampH, bacA, hns,* and *crp*) were detected in 97%–100% of the genomes.

Among other resistance determinants, aminoglycoside-modifying enzymes were the most abundant class. The *aac(6')-Iy* gene was dominant, present in 77.3% of genomes, followed by *aac(6')-Iaa* (22.7%) and *ant(3'')-IIa* (7.4%). Other *aac, ant, aph,* and *aadA* variants occurred at lower frequencies, indicating a broad but uneven distribution of enzymatic aminoglycoside resistance mechanisms.

β-Lactam resistance genes were moderately represented. The *bla_TEM-1_* gene was the predominant β-lactamase (4.5%), while extended-spectrum β-lactamases (ESBLs) such as *bla*_CTX-M-55_, *bla*_CTX-M-65_, and *bla*_CTX-M-9_ were detected at low frequencies (<1%). AmpC-type β-lactamases, including *bla*_CMY-59_ (1.9%) and *bla*_DHA-1_ (0.4%), were also identified, whereas carbapenemase-like genes (*bla*_CARB-3_ and *bla*_LAP-2_) occurred sporadically. Tetracycline resistance was also observed, primarily driven by *tet(A)* (9%) and *tet(B)* (5.6%), with minute detection of *tet(C*) and *tet(M*). Sulfonamide and trimethoprim resistance genes were also frequent, including *sul1* (5.5%), *sul2* (4%), *sul3* (2.9%), and *dfrA* variants such as *dfrA12* (1.6%) and *dfrA14* (1.9%). Collectively, these gene families indicate substantial potential for co-trimoxazole resistance among the isolates.

Phenicol resistance was moderately prevalent, with *floR* (5.2%), *cmlA1* (1.9%), and *catI* (0.6%) representing the major determinants. Fosfomycin resistance genes were notable, with *fosA7* (7.9%) leading, followed by *fosA3* and *fosA4* at lower frequencies. In contrast, macrolide-lincosamide-streptogramin (MLS) resistance genes (*mphA*, *lnuG*, *mefB*, and *ermB*) were infrequent (<1%–1.3%).

Fluoroquinolone and colistin-resistance determinants were comparatively rare. The plasmid-mediated quinolone resistance gene, *qnrS1* (3.3%), was the most frequent within its class, followed by minor occurrences of *qnrB* variants and *aac(6')-Ib-cr* (0.1%). Mobilized colistin resistance (*mcr*) genes were few but of significant concern. *mcr-9* was identified in serogroup B (1.7%) and I (3.3%), while *mcr-1* and *mcr-5* were each detected in <1% of the isolates. The detection of *mcr-9* suggests early-stage HGT events and highlights the need for enhanced genomic surveillance to prevent the spread of colistin resistance (20).

### Whole genome-based virulome profiling

**Fig 6:**
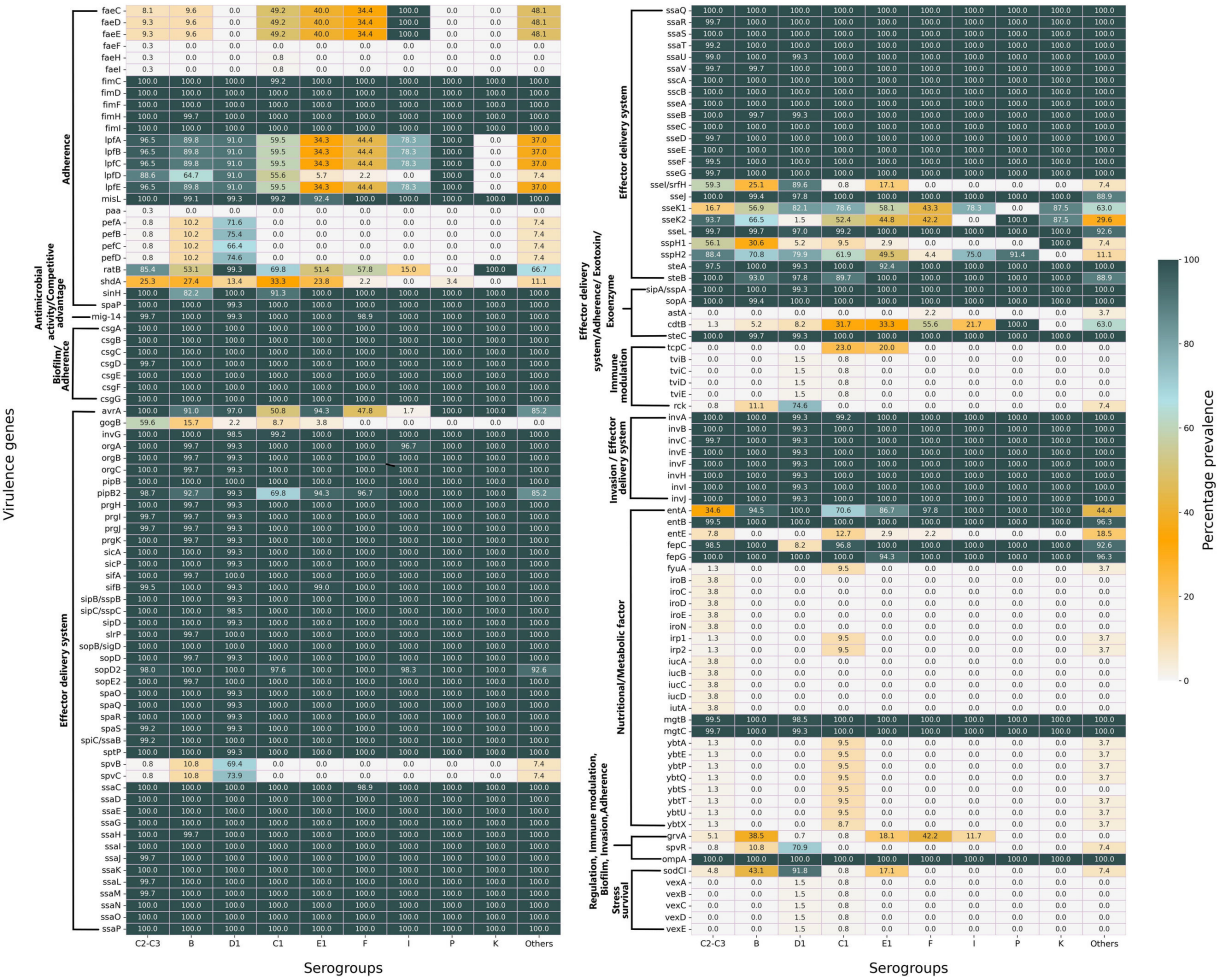
Heatmap illustrating the percent prevalence of virulence-associated genes across different serogroups: the *x*-axis represents bacterial serogroups, while the *y*-axis lists specific virulence genes. The color bar indicates percentage prevalence, ranging from 0% (light beige) to 100% (dark teal).

The comprehensive virulome analysis of the data set revealed distinct patterns in the distribution of virulence factors across multiple *Salmonella* serogroups. The data set revealed a clear dichotomy between highly conserved core genetic determinants that support pathogenicity and AMR, and a set of variably distributed accessory genes that appear to define serogroup-specific adaptability, exposure history, and ecological specialization in the top serogroups, which represent 95% of the data set (Fig. 6).

There is a high level of conservation among the core virulence factors, primarily associated with *Salmonella* pathogenicity islands (SPI-1 and SPI-2). These islands encode the critical molecular machinery required for host cell invasion, intracellular survival, and systemic infection (21, 22).

Genes associated with invasion and type III secretion system 1 (T3SS-1), including *invA*, *invC*, *invH*, *prgH*, *prgI*, *sipA*, *sipC*, and *sopB/E*, were present across all serogroups. The genes *invA* and *invC* were detected in almost all genomes, confirming their indispensable role in mediating bacterial entry into host epithelial cells. The structural components *prgH* and *prgI* of the secretion apparatus exhibited high presence (99.7%), with slightly lower frequencies in serogroup B and the miscellaneous serogroups. The key effectors mediating cytoskeletal rearrangement, *sipA* and *sipC* (23), together with *sopB* and *sopE*, were uniformly detected across all serogroups (100%), including B and C2-C3. The second pathogenicity island, housing *ssa* and *sse* gene families responsible for intracellular survival and systemic dissemination, was found to be almost entirely conserved. The genes *ssaB*, *ssaE*, *ssaR*, *ssaT*, and *ssaU* demonstrate ≥98%–100% recovery across all serogroups, underscoring their essential role in type III secretion system 2 (T3SS-2) assembly (24).

The conservation of the *sseA-sseG* operon across *Salmonella* serovars (99%–100%) highlights its essential role in pathogenesis (25). The translocon proteins SseB, SseC, and SseD are required to establish the vacuolar niche (26), while the effectors SseF and SseG mediate *Salmonella*-containing vacuole positioning near the Golgi network, thereby collectively ensuring immune evasion (27).

In contrast to the conserved SPI genes, several accessory virulence determinants exhibit serogroup-specific variability, indicating adaptive evolution or ecological niche specialization. The genes *mgtC* and *spiC*, involved in intracellular magnesium regulation (28) and macrophage survival (29), show consistently high recovery in all serogroups. This suggests enhanced intracellular adaptation in high-virulence serogroups. In our observations, *sopB* and *sopE*, which mediate host signal modulation (30), were also highly prevalent across all serogroups, indicating strong conservation and retention of these effector genes. Subtle yet consistent differences in recovery patterns were observed for *prgI* and *prgH*, suggesting lineage-specific variation in the secretion apparatus. Drastic serogroup-specific differences in the distribution of virulence genes were evident at a few key loci. The most striking distinction was observed in serogroup D1, which uniquely harbored the *spvB*, *spvC*, and *spvR* plasmid virulence genes along with the entire *pefA*, *pefB*, *pefC*, and *pefD* fimbrial operon. These genes were present at low or moderate levels across all other serogroups, highlighting a strong enrichment of plasmid-associated virulence in D1. Similarly, the *faeC*, *faeD*, and *faeE* adhesin genes have been conserved in serogroup I, representing a lineage-specific fimbrial specialization compared with other serogroups. The *sodCI* oxidative stress gene showed a dual enrichment pattern in serogroups B (43.1%) and D1 (91.8%), suggesting a shared enhancement of systemic infection potential in these groups. The *iroB*, *iroC*, *iroD*, *iroE*, and *iroN* iron acquisition genes, along with *irp1*, *irp2*, and the *iucA*, *iucB*, *iucC*, *iucD*, and *iutA* aerobactin system, were minimally conserved but showed representation in serogroups C2-C3 and C1, indicating that siderophore-mediated iron uptake is more prominent in certain invasive serogroups. The *gogB* effector gene, involved in host immune modulation, was present at moderate levels in serogroups C2-C3 (59.6%) but was absent or present at low levels elsewhere, indicating a lineage-specific adaptation pattern. The genes *ssel/srfH*, both SPI-2-encoded effectors, showed clear quantitative variation: they were present in 59.3% of C2-C3 genomes, 89.6% of D1 genomes, and only 25.1% of B genomes, highlighting differential retention of virulence factors across serogroups. The *tcpC* gene, which disrupts host innate immune signaling in the toll-like receptor pathway (31), was found only in serogroups C1 (23%) and E1 (20%). In contrast, the *cdt* (cytolethal distending toxin) gene cluster was completely absent in serogroup K, suggesting a loss or lack of acquisition of this toxin module in that lineage. The *tvi*, *vex*, and *ybt* loci were absent or present only minimally across nearly all serogroups, indicating limited distribution and a likely absence from the dominant *Salmonella* lineages in the data set.

Comparative analysis across *Salmonella* subspecies revealed a highly conserved core virulence repertoire accompanied by pronounced subspecies-specific diversification (Fig. S2: https://github.com/Viraj-105/Environmental_Salmonella_Genomics). Genes encoding type I fimbriae (*fim*), curli biogenesis (the *csg* operon), and the SPI-1/SPI-2 type III secretion systems were nearly ubiquitous, underscoring strong evolutionary constraints on functions essential for host colonization and intracellular survival. In contrast, accessory virulence determinants exhibited substantial heterogeneity. The adhesion-associated gene *sinH* was conserved in *S. enterica* and *S. salamae* but was completely absent in *S. diarizonae*, whereas another adhesin-related gene, *paa*, was selectively retained at high frequency in *S. salamae*. Non-core effector genes (*sseK1/2*, *steA/B*, and *sopA*), primarily involved in host interaction and effector delivery, showed reduced prevalence or complete loss in non-*enterica* subspecies, consistent with lineage-specific modulation of host-pathogen interactions. Notably, the motility-associated gene *fliG* was detected exclusively in *S. diarizonae*. In addition, metabolic and nutritional fitness loci, particularly iron acquisition systems (the *iuc*, *irp*, and *ybt* operons), displayed divergent retention patterns, with broader conservation in *S. diarizonae* and selective loss in *S. salamae*.

### Plasmidome analysis and integron landscape

**Fig 7:**
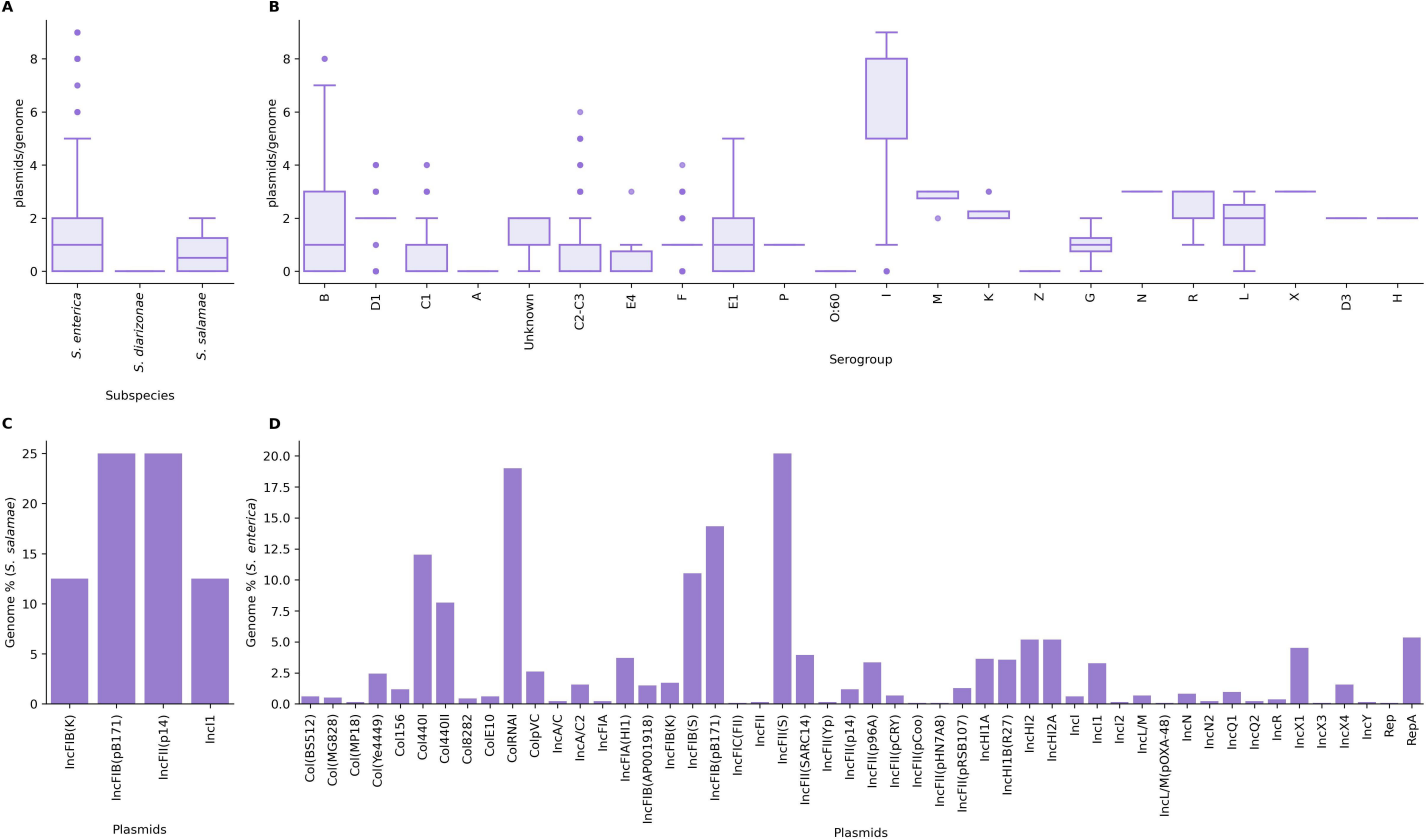
Comparative analysis of plasmid repertoire and abundance across *Salmonella* lineages. (**A**) Box plot illustrating the distribution of plasmids per genome across three *Salmonella* subspecies: *S. enterica*, *S. diarizonae*, and *S. salamae. S. enterica* displays the widest range and the highest median number of plasmids. (**B**) Box plot showing the number of plasmids per genome across diverse *Salmonella* serogroups. The distribution highlights significant variability in plasmid carriage among different serogroups. (**C**) Bar chart displaying the prevalence of plasmids identified within *S. salamae* genomes. The *y*-axis represents the percentage of genomes containing each specific plasmid type. (**D**) Bar chart displaying the percent prevalence of a wide range of plasmid types identified within *S. enterica* genomes.

*In silico* plasmidome analysis of genomes revealed a highly diverse and unevenly distributed plasmid pool (Fig. 7). When grouped by subspecies, *S. enterica* exhibited the highest plasmid count per genome, followed by *S. salamae*, while no plasmids were detected in *S. diarizonae*. Additionally, no plasmids were identified in the clinical reference genomes. Zooming in on subtypes, serogroup I exhibited the highest plasmid burden, while groups B, C1, and D1 showed lower plasmid carriage, with D1 having limited plasmid diversity despite the higher share in the population. Moderate plasmid presence is observed in M, L, and R, whereas P, H, and X contain few or no plasmids. The incompatibility plasmids represent the largest group, accounting for 64.4% of all identified plasmids. Colicinogenic plasmids formed the second largest group (31.9%), while replication plasmids were the least represented (3.6%). The most prevalent plasmids in *S. enterica* were IncFII(S), ColRNAI, and IncFIB(pB171). Less frequent but significant plasmids include IncI1, IncHI2, and IncA/C2, often associated with MDR gene transfer (32). *S. salamae* showed a narrower plasmid range dominated by the IncF type plasmids. Class 1 integrons were detected in 131 genomes, whereas class 2 integrons were present in only nine genomes, indicating a limited but noteworthy mobilome-associated resistance potential across the data set. No transposons were identified under the applied detection criteria. However, this absence may reflect the limitations in database coverage, sequence assembly quality, or detection sensitivity rather than true absence. Therefore, the contribution of transposon-mediated HGT in this collection cannot be conclusively ruled out.

### Genomic feature enrichment analysis

Genomic enrichment analysis of 1,399 isolates revealed pronounced serogroup-specific signatures encompassing AMR, virulence determinants, and plasmid replicons. The strongest enrichment was observed in serogroup B, which harbored the most significantly enriched features (*n* = 48), including 23 AMR genes. Notable examples include *fosA7* (OR = 88.1, *q* < 10^−58^), the multidrug efflux regulator *mdtK*, and the copper resistance (*pco*) operon, collectively indicating a marked genomic predisposition toward antimicrobial and chemical stress tolerance.

In contrast, serogroup C2-C3 (44 features), D1 (27 features), and M were primarily characterized by virulence-associated loci. Serogroup D1 showed strong enrichment for plasmid-encoded fimbrial (*pef*) and *Salmonella* plasmid virulence (*spv*) operons (OR >60, *q* < 10^−^⁷⁰), consistent with the high prevalence (75.4%) of the IncFIB(S)_1 replicon in this serogroup. Similarly, serogroup C2-C3 was distinguished by enrichment of the stealth siderophore cluster *iroBCDEN*, while serogroup M exhibited strict enrichment of the *fae* (K88) fimbrial operon, highlighting distinct strategies for host-niche adaptation.

Additional serogroup-specific patterns were observed, including exclusive enrichment of the yersiniabactin (*ybt*) siderophore system in serogroup O:60 (*n* = 11 genes; *fyuA, irp1, irp2, ybtA, ybtE, ybtP, ybtQ, ybtS, ybtT, ybtU,* and *ybtX*; 100% prevalence) and strong association of serogroup I with the IncFII(SARC14)_1_SARC14 plasmid replicon (OR = 577.9). Across the data set, 107 biomarkers displayed infinite odds ratios, reflecting strict serogroup restriction relative to the background population. The cytolethal distending toxin gene *cdtB* was identified across six serogroups (C1, E1, F, M, O:60, and P), reaching fixation in the latter three. Collectively, these findings indicate that while certain virulence factors are broadly distributed, serogroup-specific genomic architectures are largely shaped by high-effect-size, serogroup-restricted elements, particularly MGEs, which likely contribute to ecological specialization and niche adaptation. Detailed genome feature-serogroup associations, including odds ratios and FDR-adjusted *q*-values, are provided in Table S4 (https://github.com/Viraj-105/Environmental_Salmonella_Genomics).

### Convergence of virulence and AMR on plasmid replicons

A total of 2,131 gene-plasmid associations were identified, comprising 1,800 virulence factors (VFs) and 331 AMR genes, indicating a predominance of virulence determinants on plasmid-borne accessory genes. Notably, 15 hybrid plasmids co-harbored VF and AMR genes on a single plasmid backbone, reflecting a key convergence of pathogenicity and resistance traits. These hybrids were primarily associated with IncFIB(K)_1_Kpn3 (*n* = 5), IncI1_1_Alpha (*n* = 4), IncQ2_1 (*n* = 3), and IncFIB(AP001918)_1 (*n* = 3 each), with conserved gene pairings such as *sseK2-mdtABC-baeR*, *sinH-acrD*, and *iroBCDEN-APH(3'')-Ib/APH (6)-Id*.

Plasmids served as major reservoirs of AMR and virulence genes. Colicinogenic plasmids (31.9%) were frequently associated with *qnr*S1 on the ColRNAI and Col440II replicons, alongside *tet(A)*, *sul2*, and aminoglycoside resistance genes. Larger IncHI1A/IncHI1B plasmids carried MDR clusters, while IncF plasmids (notably IncFIB(S) and IncFII(S)) served as key virulence hubs harboring *pef*, *spvBCR*, and *rck* across 168 isolates. Importantly, hybrid IncFIB(K) plasmids co-localizing MDR genes [e.g., *dfrA14*, *sul1*, and *tet(A)*] with the *ybt* siderophore system highlight the emergence of high-risk, hypervirulent MDR lineages. Overall, AMR genes were plasmid-associated in 108 isolates and virulence factors were so in 168 isolates, underscoring plasmids as central drivers of co-selection and dissemination. The distribution of AMR genes and virulence genes across plasmid replicons is detailed in Table S3 (https://github.com/Viraj-105/Environmental_Salmonella_Genomics).

### Ecological association of genomic features and population structure

To investigate ecological structuring, associations between genomic features and isolation sources (surface water, soil, and host associated) were evaluated using the statistical framework described in Materials and Methods. All reported associations were statistically significant after FDR correction.

Host-associated isolates exhibited a markedly higher burden of AMR determinants than their environmental counterparts. Prominent resistance genes included *tet(A)* (18%), *ant(3'')-IIa* (16.3%), and *floR* (15.5%), accompanied by substantial enrichment of plasmid replicons such as IncHI2 (22%) and IncFII(SARC14) (19.2%), both of which were rare or absent among environmental isolates. Virulence-associated gene content likewise demonstrated clear ecological structuring. Host-associated genomes were significantly enriched for *sodCI* (53.1%), *sseK1* (77.6%), and the plasmid-encoded *pef* fimbrial operon. Furthermore, the *fae* fimbrial operon (including *faeD/E*) and the salmochelin siderophore cluster (*iroBCDEN*) showed strict host association, reaching prevalence up to 42.9%, while being virtually undetected in soil-derived isolates. In contrast, the *cdtB* gene was disproportionately represented in surface water (30.6%) and soil (12.2%) isolates.

These functional differences were accompanied by distinct niche-specific serovar distributions. The serovar Newport was predominantly associated with soil (46%) and surface water (19.8%), whereas Enteritidis (19.5%), Yoruba (18.3%), Typhimurium (13.2%), and Heidelberg (11.7%) were enriched in host-associated sources. At the serogroup level, serogroup B predominated in host-associated isolates (37.4%), while serogroups C2-C3 dominated soil (55.4%) and surface water (28.1%) environments.

Table S5 (https://github.com/Viraj-105/Environmental_Salmonella_Genomics) depicts ecological associations and genomic feature enrichment statistics in the context of our global collection of *Salmonella* genomes, including those of newly cultured isolates from the environmental samples.

## DISCUSSION

Genomic surveillance of pathogens is an important component of the One Health framework, providing critical insights into the ecological reservoirs where versatile bacteria such as *Salmonella* evolve, persist, and develop AMR at the human-animal-environment interface (3, 33, 34). Environmental compartments remain systematically under-sampled despite their central role in shaping pathogen population structure and serving as early indicators of emerging resistance and virulence traits. By integrating phenotypic characterization of Indian environmental isolates with large-scale comparative genomics, this study establishes a distinct portfolio of environmental *Salmonella* within the global evolutionary scenario of diarrheal disease pathogens while also exposing key surveillance gaps.

The comparative analyses in this study relied primarily on open-source genomic data sets, which are indispensable for global-scale inference but remain heavily skewed toward high-income countries, particularly the United States. This overrepresentation likely reflects sustained investment in coordinated surveillance systems such as those led by the CDC (35), which generate high-resolution longitudinal genomic data. While these resources enable robust comparative analyses, they also introduce geographical bias, limiting inference for regions with high environmental exposure but limited sequencing capacity (36). Within this context, the inclusion of Indian environmental isolates provides valuable representation from a setting characterized by intense human-animal-environmental interactions and fragmented environmental monitoring.

Phenotypic analysis of the in-house isolates revealed a substantial proportion exhibiting a MDR phenotype, accompanied by widespread biofilm-forming capacity, suggesting enhanced environmental persistence and resilience. Biofilm formation likely facilitates survival across aquatic and soil matrices while promoting the retention and exchange of resistance determinants (37). Observed resistance patterns, including reduced susceptibility to clinically relevant antimicrobials, may reflect anthropogenic selective pressures, such as contamination from human sewage, healthcare effluents, agricultural runoff, or indirect exposure to antimicrobials, thereby reinforcing the role of environmental reservoirs as early-warning systems for the evolution of resistance (38, 39).

Environmental contexts play a multifaceted role in shaping *Salmonella* population structure, extending beyond passive reservoirs to active sites of selection and evolutionary diversification. Anthropogenically influenced environments, including wastewater systems and agricultural settings, impose sustained selective pressures that promote the maintenance and dissemination of resistance and virulence-associated traits (40–42). While HGT represents a major driver of genomic plasticity, additional mechanisms, including homologous recombination, gene loss, and regulatory adaptation, also contribute to lineage diversification and ecological fitness (43). Importantly, these environments should be distinguished not only as reservoirs of genetic diversity but also as potential amplification niches and transmission interfaces, where selective enrichment and population expansion may facilitate downstream dissemination into host-associated systems. Together, these perspectives provide a more nuanced framework for understanding the ecological and evolutionary dynamics underlying the distribution of *Salmonella* lineages.

Whole-genome comparison indicates no distinct geographical segregation of in-house isolates within an interspersed global phylogenetic framework. While this pattern may reflect shared ancestry or environmental mixing, it does not constitute definitive evidence of transboundary dissemination, as it may also arise from convergent evolution or the persistence of ancestral lineages. The lack of geographic clustering likely reflects sampling bias, with global genomic data sets disproportionately derived from high-income regions, particularly the United States, thereby limiting the resolution of regional evolutionary dynamics. Pangenome analysis highlighted the pronounced genomic plasticity of environmental *Salmonella*, characterized by a conserved core genome and a large, dynamic accessory gene pool shaped by HGT and niche adaptation. While evidence of recent clonal expansion was observed in certain lineages, deep-branching clades suggest long-term persistence within environmental reservoirs rather than recent spillover.

Resistome analyses revealed a conserved intrinsic resistance backbone across serogroups and subspecies, alongside heterogeneous acquisition of clinically significant resistance genes. Although detected at low frequency, the presence of ESBLs, AmpC enzymes, and colistin resistance markers raises concern regarding the silent circulation of “last-line” antibiotic resistance determinants in environmental settings. In parallel, virulome and plasmidome profiling demonstrated strong conservation of core pathogenicity islands, in contrast to serogroup- and lineage-specific distributions of accessory virulence factors and MGEs, reflecting distinct ecological niches and selective pressures.

Serogroup-level enrichment patterns reveal clear functional differentiation within *Salmonella* populations. The enrichment of AMR determinants in serogroup B suggests selective pressures favoring resistance-associated traits, whereas serogroups C2-C3 and D1 are primarily characterized by virulence-associated loci, including plasmid-encoded *pef* and *spv* operons. The exclusive presence of the yersiniabactin (*ybt*) system in serogroup O:60 and the strong association of specific plasmid replicons with defined serogroups further highlight the role of MGEs in shaping genomic diversity. Overall, the presence of high-effect, serogroup-restricted features supports the idea that ecological specialization is driven by lineage-associated genetic modules rather than uniformly distributed traits.

In our observation, publicly available genomic data mostly represent isolates from only 25 of the world’s 195 countries, with a pronounced bias toward North America. This uneven representation likely influences observed trends in lineage prevalences, resistance profiles, and population structure. Environmental sampling within India was geographically constrained and may not capture broader spatial or seasonal heterogeneity. Phenotypic resistance data were available only for in-house isolates, restricting genotype-phenotype correlations across the global data set. Although multiple intrinsic resistance determinants were detected, these primarily encode efflux pumps and regulatory elements whose effects depend on their expression or on environmental cues. Consequently, antimicrobial susceptibility cannot be directly inferred from the presence/absence of genes alone, highlighting the need for functional, expression-based, and mutational screens/analyses to achieve meaningful genotype-phenotype correlations. Still, these findings provide a foundational view of the core resistome and regulatory landscape in global *Salmonella* isolates. Similarly, although biofilm-associated genes (e.g., *csg*, *bcs*, and quorum-sensing loci) were widely present, their presence does not reliably predict phenotype, which is shaped by regulatory networks and environmental cues. Furthermore, our findings reinforce a much-needed snapshot of the core biofilm-associated potential of environmental *Salmonella* isolates.

The study shows that environmental *Salmonella* populations function not as passive reservoirs but as active evolutionary arenas where persistence, virulence, and AMR are continuously shaped and exchanged. Addressing geographical gaps in analyzing pathogens and their spillovers in the environmental niches, particularly in underrepresented regions, is essential for strengthening One Health strategies. Therefore, enabling more equitable and earlier detection of emerging public health threats is the need of the hour in the context of integrated pathogen epidemiology based on genomic surveillance.

### Conclusions

Environmental *Salmonella* and their complex and dynamic occurrence were dissected through integrated genomic and phenotypic approaches in this study while underscoring their considerable genetic diversity and adaptability across global ecosystems.

Our findings demonstrate that environmental *Salmonella* maintain a stable core of virulence-associated genes while exhibiting substantial variability in accessory genes associated with resistance and virulence. The dominance of intrinsic efflux systems, the widespread capacity for biofilm formation, and the presence of early-stage resistance markers, such as intermediate fluoroquinolone resistance and low-frequency *mcr* genes, underscore the role of environmental reservoirs as an early marker of emerging AMR.

The enrichment of virulence genes in specific serogroups and the distinct plasmid profiles we observed reinforce the significance of HGT in driving ecological fitness and niche adaptation. Together, the study underscores an urgent need for integrated environmental surveillance systems that incorporate genomic monitoring of resistance and virulence traits in bacterial pathogens. These systems are essential for tracking the emergence of AMR and identifying potential spillover events. The accumulation of resistance and virulence factors in environmental *Salmonella* isolates could be symptomatic of broader systemic failures, including unmanaged waste, unsustainable antibiotic use in agriculture, and inadequate environmental monitoring. Needless to mention, addressing these challenges requires a One Health approach that bridges the human, animal, and environmental domains.

Incorporating environmental genomics into global and national AMR action plans (44, 45), enhancing wastewater treatment infrastructure, and regulating antibiotic use in food production are critical steps to mitigate the environmental amplification of resistance. A holistic, cross-sectoral strategy that includes the environment as an active participant in the AMR landscape is imperative to mitigate the spread of high-risk *Salmonella* lineages and protect public health. This study highlights the need for concerted global efforts to strengthen epidemiology and control measures, particularly in ecologically complex regions, to prevent the recurrent dissemination of MDR isolates of environmental *Salmonella*.

## MATERIALS AND METHODS

### Genome data acquisition and quality control

Genome data in the form of 14,262 RefSeq assemblies (complete and draft) of *Salmonella* spp. available as of July 5, 2024, were retrieved from NCBI database using the command-line interface. BioSample information from the associated metadata file was parsed in Python to identify environmental *Salmonella* genomes. To ensure high-quality genomic data, assemblies were filtered based on the following criteria: (i) no more than 200 scaffolds or contigs, (ii) GC content between 50% and 53%, (iii) genome size between 4.5 and 5.5 Mbp, and (iv) N50 value greater than 50,000 bp. Only genomes with ≥95% completeness and ≤4% contamination, as estimated by CheckM (46), were retained for downstream analyses to accommodate natural genomic variation across divergent lineages. However, non-*enterica* lineages (e.g., *S. diarizonae* and *S. salamae*) with slightly higher contamination (4.5%–7.4%) were intentionally retained. This adjustment accounts for the fact that standard marker gene sets are optimized for dominant clinical taxa (e.g., *S. enterica* subsp. *enterica*), and lineage-specific gene duplications or evolutionary divergence in rare clades can be misclassified as contamination. Given their high completeness (>95%) and core genomic integrity, these genomes were included to ensure a representative evolutionary profile of the genus. Genomes failing these broadened quality and taxonomic criteria were excluded. This filtering yielded a data set of 1,333 genomes. The assembly quality was assessed using QUAST v5.1.0 (47). In addition, 12 well-characterized reference genomes representing clinically relevant *Salmonella* serovars were included based on their complete annotations, established research use, and clinical relevance (Table S1; https://github.com/Viraj-105/Environmental_Salmonella_Genomics).

To broaden the genomic data set and capture greater ecological diversity, we isolated *Salmonella* from 506 environmental samples collected across multiple ecological niches in and around Hyderabad, India. Aliquots from each sample were enriched in Buffered Peptone Water for 24 h at 37°C, followed by selective enrichment in Rappaport-Vassiliadis Soya broth and isolation on xylose lysine deoxycholate and bismuth sulfite agar. Colonies displaying morphology consistent with *Salmonella* were sub-cultured and preserved in 50% glycerol at −20°C.

We confirmed presumptive isolates using standard biochemical assays and extracted genomic DNA with the QIAamp DNA Mini Kit (Qiagen, USA). Molecular confirmation targeted the *invA* gene (284 bp), as described previously (48, 49). PCR products were visualized on 1.5% agarose gels under ultraviolet illumination, using *S. enterica* subsp. *enterica* serovar Typhimurium ATCC 14028 as the positive control.

All PCR-confirmed isolates were subsequently subjected to WGS, using both short-read and long-read platforms, followed by quality control and downstream analysis using standardized bioinformatics workflows to ensure high-quality genome assemblies. Total genomic DNA was prepared for sequencing using the Nextera XT library preparation kit (Illumina Inc., USA), and short-read sequencing was performed on the Illumina NextSeq platform with a 2 × 150 bp paired-end configuration. The quality of these reads was assessed using FastQC v0.12.1 (50), and high-quality reads were assembled *de novo* using SPAdes Genome Assembler v4.0.0 (51). The Oxford Nanopore MinION platform was employed for long-read sequencing; libraries were prepared using the SQK-NBD114-24 native barcoding kit and purified with AMPure XP beads. The prepared sequencing libraries were loaded onto an R10.4.1 flow cell (flow cell ID: FBA76412) and sequenced on a MinION Mk1B platform using MinKNOW software. Base calling was performed using Dorado with the high-accuracy model (400 bp). The generated reads were systematically evaluated for quality metrics and length distribution using NanoPlot (52). *De novo* assembly of Nanopore reads was conducted using Flye v2.9.5 (53). Furthermore, the quality of the in-house assemblies was assessed using QUAST v5.1.0 (47). In total, 50 genomes (ENV_INHO_0001 to ENV_INHO_0050) were generated and assembled from Illumina short reads, while four (ENV_INHO_0051 to ENV_INHO_ 0054) were from ONT long reads. Each genome was assembled exclusively from a single-platform data set, with no hybrid assemblies.

The final data set used for downstream comparative genomic analyses comprised 1,399 high-quality genomes, including 1,333 publicly available environmental assemblies, 12 clinical reference genomes, and 54 newly sequenced in-house genomes (Table S2: https://github.com/Viraj-105/Environmental_Salmonella_Genomics).

### AST and biofilm formation assay

AST of the in-house isolates was done using the Kirby-Bauer disk diffusion method on Mueller-Hinton agar plates (54). The results were interpreted according to Clinical and Laboratory Standards Institute guidelines (55). Isolates exhibiting resistance to three or more classes of antibiotics were classified as MDR. The standard crystal violet assay was used to assess biofilm formation, as previously described (56). The positive control was *S. enterica* subsp. *enterica* serovar Typhimurium (ATCC 14028), and *E. coli* DH5α was used as the negative control. Based on OD_570_ values relative to the negative control, biofilm formation was categorized into: weak (SBF ≤ 0.1), moderate (0.1 < SBF ≤ 0.5), and strong (SBF > 0.5). The SBF was calculated using the standard formula (56).

### Generation of pangenome data and phylogenetic analysis

The final set of genomes (*n* = 1,399), including in-house genomes, was annotated using Prokka (57). The resulting GFF files were subsequently used as input for pangenome analysis with Panaroo v1.5.1 (58) in strict mode, employing MAFFT (59) for core-genome alignment. The concatenated core gene alignments were subsequently used to infer a maximum-likelihood phylogenetic tree using IQ-TREE (v3.0.1) (60) with the generalized time-reversible (GTR) model of nucleotide substitution, and a Gamma distribution of rate heterogeneity (GTR + G), applying 1,000 ultrafast bootstrap replicates. The resulting phylogenetic tree was visualized using iTOL (Interactive Tree of Life v7) (61).

### *In silico* typing: serogroup, serovar, and MLST

*Salmonella In Silico* Typing Resource (SISTR) (62) was used to predict serogroups and serovars of the *Salmonella* genomes under study. MLST was performed using the *in silico* MLST tool (https://github.com/tseemann/mlst) (63), employing the senterica_achtman_2 scheme. To determine STs, an entire data set comprising environmental genomes and clinical reference strains was analyzed based on the allelic profiles of seven housekeeping loci (*aroC, dnaN, hemD, hisD, purE, sucA,* and *thrA*).

### Whole genome-based resistome, virulome, and mobilome profiling

AMR encoding genes, virulence genes, and plasmid replicons were predicted across the final data set of 1,399 genomes using ABRicate v1.0.1 (https://github.com/tseemann/abricate), with a minimum identity and coverage threshold of ≥80%, selected as standard practice in comparative genomics to balance sensitivity and specificity across diverse *Salmonella* lineages. The Comprehensive Antibiotic Resistance Database (CARD; 2,631 sequences) (64) and the Virulence Factor Database (VFDB; 2,597 sequences) (65) were used as reference databases for AMR and virulence gene prediction, respectively. Similarly, the PlasmidFinder database (460 sequences) (66) was used to identify plasmid replicons. All ABRicate-integrated databases were accessed on 15 December 2025. Integrons and transposon regions were detected using the standalone version of BacAnt v3.3.1 (67) with default parameters.

### Genomic feature enrichment and statistical analysis

Outputs from the CARD, the VFDB, and PlasmidFinder were integrated with *Salmonella* serogroup assignments. Genomic summary files were standardized by extracting unique sample identifiers from file base names, and binary presence-absence matrices were generated, encoding features as present (1) or absent (0) based on default pipeline thresholds.

Serogroup-specific enrichment analyses were performed by comparing feature frequencies within a given lineage against a background of other isolates. For each serogroup combination, a 2 × 2 contingency table was constructed, and statistical significance was assessed using two-sided Fisher’s exact tests (68). Effect sizes were estimated using odds ratios (ORs), with infinite ORs assigned to serogroup-exclusive features. *P*-values were adjusted for multiple testing using the Benjamini-Hochberg FDR (69), and features with adjusted *q* < 0.05 were considered significantly enriched. All data processing and statistical analyses were conducted in Python (v3.13.12) using the pandas, SciPy (70), and statsmodels libraries. The results were compiled into a comprehensive enrichment table and summarized by functional category, including AMR genes, virulence factors, and plasmid replicons for each serogroup.

### Plasmid co-localization analysis

To assess the physical co-occurrence of functional genetic elements, we performed a systematic plasmid co-localization analysis. Reconstructed plasmid contigs were cross-referenced with AMR and virulence gene annotations to identify specific genetic linkages. Plasmid replicons were identified using PlasmidFinder, while AMR and virulence genes were identified using the CARD and VFDB databases, respectively. A custom bioinformatics pipeline was used to summarize gene-plasmid associations by matching features to shared contig identifiers. This approach enabled the identification of plasmid-borne resistance and virulence elements. Furthermore, we screened for hybrid plasmids, defined as single contigs co-harboring at least one plasmid replicon, one AMR gene, and one virulence determinant, to identify plasmid backbones capable of transmitting both traits simultaneously.

### Statistical methods relevant to genomic features and ecological and lineage association analyses

Isolation sources were standardized into ecological categories using keyword-based metadata parsing, defining surface water, soil, host-associated environments, and other environmental groups, with clinical isolates classified separately as host clinical isolates. For downstream analyses, host-associated environmental and clinical isolates were combined into a unified host*-*associated category where appropriate.

All *P*-values were corrected using the Benjamini-Hochberg FDR, with adjusted *P* < 0.05 considered significant. All analyses were performed in Python (v3.10) using pandas, SciPy, and statsmodels.

Associations between genomic features (AMR genes, virulence factors, and plasmid markers) and ecological categories were assessed using χ^2^ tests of independence on presence-absence contingency tables, restricted to features exhibiting variation across groups. Feature prevalence was calculated as the proportion of genomes carrying each feature within a given category, and lineage-ecology associations (serovars and serogroups) were evaluated using the same framework. Analyses were limited to categories with ≥5 observations (excluding undefined metadata) in the context of the entire genome data.

## Data Availability

All genome assemblies generated in this study have been deposited in the NCBI Genome Database under BioProject accession number PRJNA1231798. Individual accession numbers are provided in Table S2. All reference genomes were retrieved from the NCBI RefSeq database. All supplemental figures and tables have been deposited in the GitHub repository at https://github.com/Viraj-105/Environmental_Salmonella_Genomics.
